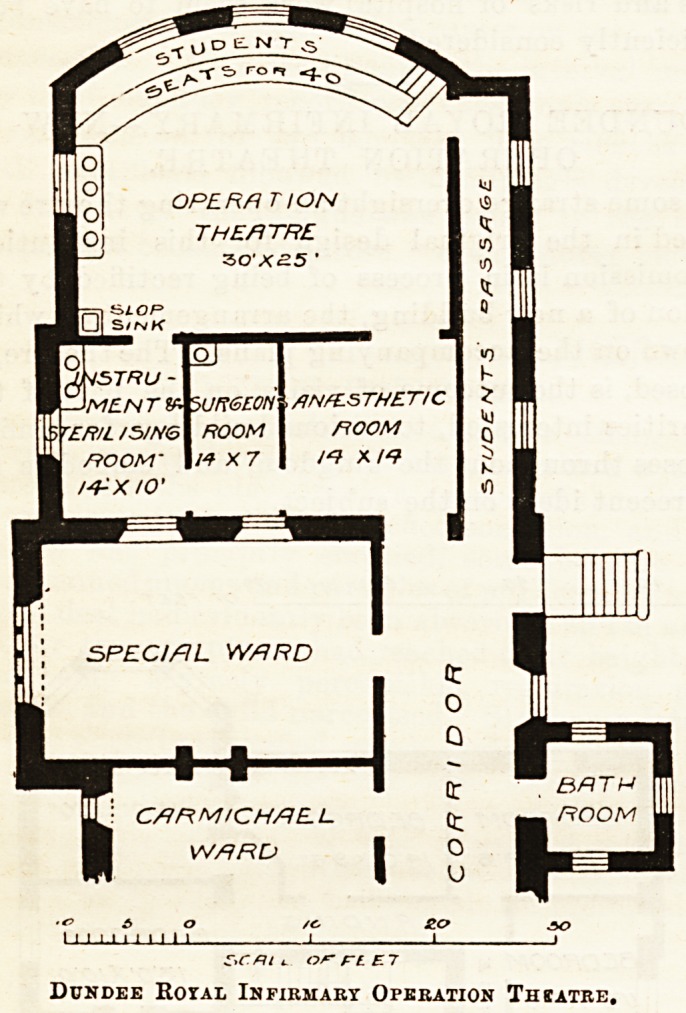# Dundee Royal Infirmary—New Operation Theatre

**Published:** 1895-07-06

**Authors:** 


### DUNDEE ROYAL INFIRMARY.—NEW OPERATION THEATRE.

By some strange oversight an operating theatre was omitted in the original design for this institution. This omission is in process of being rectified by the erection of a new building, the arrangement of which is shown on the accompanying plans. The theatre, as proposed, is the outcome of visits, on the part of the authorities interested, to various buildings for similar purposes throughout the kingdom, and embodies the most recent ideas on the subject.

The patients are brought into the theatre through the anæsthetic room, with which the surgeon's room communicates, and in which they are prepared for operation. A sterilising room for the treatment of instruments, dressings, &c., by steam, is placed in communication with the surgeon's room and also with the theatre.

The lighting of the theatre is effected by a large circular window towards the north, as well as by a top light. It is floored with terrazzo, its walls are tile-lined, and arrangements are made for sluicing it down with water or disinfectants. It is heated by steam pipes and radiators, and every precaution has been taken to keep aseptic principles in view, both in the matter of fittings and finishings. A separate entrance and sitting accommodation for a small number of students are provided.

It is assumed that the windows shown on the plan sent to us for publication between the special ward and the rooms adjoining the theatre will be blocked, or represent lights above the roof of the new buildings.

Dundee Royal Infirmary Operation Theatre.

## Figures and Tables

**Figure f1:**